# Ciliochoroidal detachment after Ahmed glaucoma valve implantation: a retrospective study

**DOI:** 10.1186/s12886-019-1060-y

**Published:** 2019-02-06

**Authors:** Lin Fu, Yau Kei Chan, Li Nie, Qi Dai, Zhenbin Qian, Kendrick Co Shih, Jimmy Shiu Ming Lai, Rong Huang, Weihua Pan

**Affiliations:** 1 Affiliated Eye Hospital, School of Ophthalmology and Optometry, Wenzhou Medical University, Zhejiang, China; 2 Department of Mechanical Engineering, Faculty of Engineering, University of Hong Kong, Hong Kong, Hong Kong SAR; 3 Department of Ophthalmology, LKS Faculty of Medicine, University of Hong Kong, Hong Kong, Hong Kong SAR; 4 Department of Ophthalmology, LKS Faculty of Medicine, University of Hong Kong, Hong Kong, Hong Kong SAR; 5 MOE-Shanghai Key Laboratory of Children’s Environmental Health, Xinhua Hospital, Shanghai Jiao Tong University, Shanghai, China

**Keywords:** Ciliochoroidal detachment, Ahmed glaucoma valve, success rate

## Abstract

**Background:**

To investigate the occurrence of ciliochoroidal detachment (CCD), its risk factors and its impact on the success rate after Ahmed glaucoma valve (AGV) implantation.

**Methods:**

This is a retrospective observational study carried out at Eye Hospital of Wenzhou Medical University, Zhejiang, China. Patients with uncontrolled glaucoma who underwent AGV implantation alone or combined with phacoemulsification (AGV-Phaco) in the hospital from April 1, 2013 to July 31, 2016 were included. The preoperative and postoperative CCD was defined when the detachment between ciliary body and choroid was detected by the ultrasound biomicroscopy (UBM) and anterior segment optical coherence tomography (AS-OCT) respectively. The main outcomes included the incidence of CCD and the success rate at 6 months after surgery.

**Results:**

In total, 97 male and 56 female patients were included. CCD was observed in 92 (57.8%) eyes. The glaucoma diagnosis in the Non-CCD and CCD group included primary open angle glaucoma (21(31.3%) vs 33(35.9%)), primary angle closure glaucoma (10(14.9%) vs 13(14.1%)), secondary glaucoma (25(37.3%) vs (28(30.4%)) and so on. The preoperative median IOP (interquartile range) were 21.7(16.0,32.0) mmHg and 23.0(16.0,33.0) mmHg in the Non-CCD group and CCD group. Previous surgical history (95% confidence interval (CI), 1.24 to 13.34; odds ratio (OR) 4.06; *p* = 0.02) and shorter axial length (95% CI, 0.62 to 0.97 OR 0.78; p = 0.02) were the two risk factors of CCD. The success rate between the CCD and Non-CCD group was not significantly different (64.3% vs 62.5%, *p* = 0.86) at 6 months.

**Conclusions:**

The incidence of CCD is 57.8% after AGV surgery. Eyes with previous surgical procedure was prone to CCD occurrence and longer axial length was protective against CCD. But at 6 months postoperatively, CCD did not reduce the success rate of AGV surgery and may not be a worrisome complication.

## Background

Ciliochoroidal detachment (CCD) is a common complication after glaucoma filtering surgeries and some retinal procedures [1–5]. Its incidence ranges from 42 to 90% in the ab interno trabeculotomy, microincision vitrectomy, trabeculectomy and retinal photocoagulation surgeries. The increase in uveoscleral aqueous outflow is suggested as the mechanism of CCD. However, its relationship with postoperative intraocular pressure (IOP) is still controversial [1, 2, 6, 7]. Since the control of IOP is critical after glaucoma surgery especially in refractory glaucoma, the risk factors of CCD and its influence on the IOP after glaucoma-related surgery requires a detailed investigation.

The use of aqueous shunt is traditionally considered as the last resort of treatment for refractory glaucoma after failure of medical treatment and trabeculectomy [8, 9]. With the more promising success rate of long-term aqueous tube shunt implantation and less additional surgery required than trabeculectomy, the aqueous shunt becomes more preferred in refractory glaucoma over trabeculectomy as the surgical management [10–12]. Combined phacoemulsification and Ahmed glaucoma valve (AGV) implantation is applied in the presence of cataract [13]. CCD is not uncommon after Ahmed valve implantation [14, 15], while the investigation of CCD in AGV surgery is scarce. The purpose of this study is to display a detailed description of CCD after AGV surgery, explore the triggers and investigate its effect on the postoperative IOP. We also compare its differential impacts on the combined phacoemulsification with AGV implantation versus AGV implantation alone.

## Methods

### Study population

This is a retrospective study of patients who underwent Ahmed glaucoma valve implantation alone or combined with phacoemulsification (AGV-Phaco) in Eye Hospital of Wenzhou Medical University from April 1, 2013 to July 31, 2016. This study is in accordance to the tenets of the Declaration of Helsinki and is approved by the institutional review board of the affiliated Eye Hospital of Wenzhou Medical University. Written informed consents were provided by all patients. Patients with refractory glaucoma that failed in IOP control after multiple surgeries or under maximum medications were included in this study. And the patients with preoperative CCD, corneal endothelium decompensation, and with comorbid diseases that makes the patient not endurable for the surgery were excluded in this study. Postoperatively, in cases with hypotony, ocular ultrasound was performed and posterior choroidal detachment was found in 2 cases. They were excluded in this study.

Glaucoma patients with cataract were determined to receive AGV-Phaco when one of the following criteria was satisfied. These criteria included best-corrected visual acuity (BCVA) < 0.5, peripheral anterior chamber thickness < 1/4 corneal thickness (CT) or central anterior chamber depth < 2 mm. The other patients received AGV-alone treatment. All operations were conducted by the same experienced surgeon (WH Pan) with a standardized technique after obtaining relevant informed consent.

### Surgical procedure

The AGV implantation was modified according to previous reported literature [16]. The valve was primed by balance salt solution before implantation. Under topical anesthesia, a corneal traction was made to fix the eyeball. Then peribulbar anesthesia was performed to create a fornix-based conjunctival flap and Tenon capsule in the upper temporal quadrant. Posterior dissection of the conjunctiva and sclera was conducted and the plate was implanted to this pocket. A sponge infiltrated with mitomycin was placed on the sclera for 5 min and flushed away later. The plate was fixed by a 5–0 Prolene suture 10 mm away from the limbus.

A lateral corneal incision was created and viscoelastics was injected to maintain the anterior chamber. If this surgery was combined with phacoemulsification, a standard clear corneal phacoemulsification was performed with an artificial intraocular lens implantation and viscoelastics was left in the anterior chamber. The tube was trimmed to a length of 3 to 3.5 mm in the anterior chamber. A 23G needle track was made through an intrascleral tunnel incision and the tube was inserted along this track. The tube was ligated near the tube-plate junction to avoid postoperative hypotony by an 8–0 Vicryl suture. The Tenon capsule and conjunctival incision were repositioned to the limbus. Lastly a subconjunctival dexamethasone injection and antibiotic eyedrops were given and the surgical eye was covered by cotton gauzes.

### Clinical examinations

Baseline assessments were recorded preoperatively including IOP measurements, BCVA, axial length, corneal endothelium density to confirm the compensated function, lens status, previous surgery, and glaucoma medication score. Postoperatively, IOP was measured at day-1, CCD detected, CCD reattached and follow-up visits at week-1, month-1, month-2, month-3 and month-6. Besides, the lowest IOP during CCD was extracted for analysis. Anterior segment optical coherence tomography (AS-OCT, Visante OCT, model 1000; Carl Zeiss Meditec) was performed at day-1 and continuously during the follow-up period to assess CCD until it was resolved during the 6-month follow-up period.

### Anterior segment optical coherence tomography

The investigation of CCD was carried out using Visante OCT system at 1310 nm according to our previous published protocol [3]. The CCD was determined when there was a detachment between the ciliary body and choroid. Briefly, patients were seated in a well-lighted room with a comfortable height. The examination of 360° of the anterior segment together with ciliary body was conducted. The severity of CCD was classified into four grades including grade 0 (no CCD observed), grade 1 (slit-like; supraciliary space ≤1/2 of the ciliary body thickness), grade 2 (bandlike; supraciliary space > 1/2 of half of the ciliary body thickness), and grade 3 (obvious; supraciliary space ≥the whole thickness of ciliary body thickness). The location of CCD was recorded by quadrant count.

### Definition of success, modified success and lost rate

According to the recommendation of the World Glaucoma Association [17], the glaucoma surgery was defined as success if one of the following criteria was met in between two consecutive visits after 3 months without anti-glaucoma medications and additional interventions. These included [1] the postoperative IOP value was between 5 and 21 mmHg (5 < IOP ≤ 21 mmHg) or [2] a decrease of IOP with more than 20%. Modified success was considered when the last visit of the lost patients’ treatment outcome was included too.

### Statistical analysis

All data were analyzed using a commercially available statistical software (SPSS for Mac version24; IBM-SPSS, Chicago, IL). All categorical variables were compared by chi-square test. The numeric variables were assessed by Kolmogorov–Smirnov test for the normality of distribution. They were presented as median and inter-quartile range (IQR). The Man-Whitney *U* test was used to evaluate the non-normal variables and the normal distributed variables were assessed by Student *t* test.A logistic regression analysis was used to determine the factors associated with the CCD occurrence. Statistical significance was set at *p* value < 0.05.

## Results

**Fig. 1:**
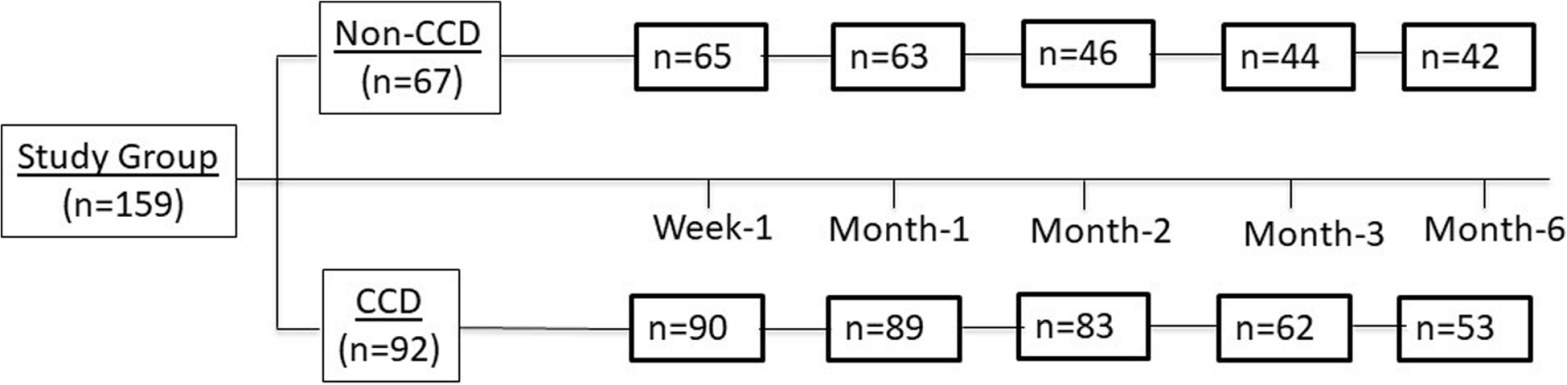
Patient Retention at 6 Months. From baseline, patients of 64% stayed in the AGV alone group and 57% stayed in the AGV-Phaco group six months after surgery

Hundred and fifty-nine eyes from 154 patients were included in this study from March 1, 2013 to July 31, 2016 with 97 males and 56 females. Among these eyes, 90 (57%) of them received AGV alone and the remaining 69 (43%) underwent AGV-Phaco (Fig. 1). At 6 months after surgery, retention rate was 64% in the AGV alone group and 57% in the AGV-Phaco group (Fig. 1).

Table 1 showed the demographic information of patients with and without CCD. The mean age was 59.4 ± 1.92 years old in the non-CCD group and 61.77 ± 1.52 years old in the CCD group without statistical significant difference. In CCD group, 62% of the eyes had previous surgical record, which is significantly more than that in non-CCD group, with only 32% of the eyes have surgical history (*p* <  0.05; Table 1). It showed no significant differences in the age, gender, diabetes, hypertension, glaucoma diagnosis, axial length, lens status, baseline medication, IOP and BCVA between CCD and non-CCD group (all *p* > 0.05; Table 1). The glaucoma diagnosis in the Non-CCD and CCD group included primary open angle glaucoma (21(31.3%) vs 33(35.9%)), primary angle closure glaucoma (10(14.9%) vs 13(14.1%)), secondary glaucoma (25(37.3%) vs (28(30.4%)), congenital glaucoma (1(1.5%) vs 3(3.3%)), mixed glaucoma (0(0%) vs 7(7.6%)) and others (10(14.9%) vs 8(8.7%)). In these two groups, the lens status consisted clear lens (8(12%) vs 6(7%)), artificial intraocular lens (IOL) (5(8%) vs 22(24%)), aphakic (3(5%) vs 2(2%)), complicated cataract (38(57%) vs 36(40%)), age-related cataract (10(15%) vs 20(22%)), traumatic cataract (1(2%) vs 3(3%)) and diabetic cataract (2(3%) vs 3(3%)). The preoperative median IOP (interquartile range) were 21.7(16.0,32.0)mmHg and 23.0(16.0,33.0)mmHg in the Non-CCD group and CCD group.

**Table 1:** Demographic information for patients in CCD and Non-CCD Group

Variable	Non-CCD Group	CCD Group	
	Total (*n* = 67)	Total (*n* = 92)	*P* value
Age, median (IQR), y	63.0 (49.0,69.0)	64.0 (54.0,73.0)	0.35
Gender, No.			0.78#
Male	43 (64%)	57 (62%)	
Female	24 (36%)	35 (38%)	
Diabetes	7 (10%)	12 (13%)	0.62#
Hypertension	25 (38%)	41 (45%)	0.40#
Glaucoma diagnosis, No (percentage)			0.26#
POAG	21 (31.3%)	33 (35.9%)	
PACG	10 (14.9%)	13(14.1%)	
Secondary	25 (37.3%)	28(30.4%)	
Congenital	1 (1.5%)	3 (3.3%)	
Mixed	0 (0%)	7 (7.6%)	
Others	10 (14.9%)	8 (8.7%)	
Axial length, median (IQR), mm	23.9 (23.1,25.1)	23.2 (22.5,24.3)	0.02*
Previous surgery, No.	22 (32%)	57 (62%)	< 0.001#
Lens status, No.			0.06#
Clear	8 (12%)	6 (7%)	
IOL	5 (8%)	22 (24%)	
Aphakic	3 (5%)	2 (2%)	
Complicated cataract	38 (57%)	36 (40%)	
Age-related cataract	10 (15%)	20 (22%)	
Traumatic cataract	1 (2%)	3 (3%)	
Diabetic cataract	2 (3%)	3 (3%)	
Baseline medication, median (IQR)	2.0 (2.0,3.0)	2.0 (1.5,3.0)	0.82*
IOP, median (IQR), mmHg	21.7 (16.0,32.0)	23.0 (16.0,33.0)	0.75*
BCVA, median (IQR)	0.6 (0.2,0.7)	0.5(0.3,0.8)	0.67^

*IQR* interquartile range, *POAG* primary open angle glaucoma, *PACG* primary angle closure glaucoma, *IOL* intraocular lens, *IOP* intraocular pressure, *BCVA* best corrected visual acuity. *Mann-Whitney *U* test, #Pearson chi square, ^Student *t* test

Postoperatively, CCD was observed in 92 (57.8%) eyes. It occurred between 1st and 18th day after surgery. The start date was significant later in AGV-Phaco group than AGV alone group (Table 2). In less than 7 days after surgery, 84.2% experienced CCD in the AGV-Phaco group while 94.2% eyes were detected with CCD in AGV alone group. The time for reposition of CCD ranged from 3rd to 180th day postoperatively. However, there was one eye in each group which CCD still did not recover till the end of our study period. No significant differences were found between the time of reposition of CCD, grading of CCD, CCD quadrant, lowest IOP during the presence of CCD, first record of IOP when CCD occurred and IOP when CCD resolved.

**Table 2:** CCD in two surgical types

	AGV-Phaco (*n* = 40)	AGV alone (*n* = 52)	*p* value
Start time, day, median (IQR)	3.0 (1.0, 4.25)	1.0 (1.0, 2.0)	0.001
< 7 days, no.	32 (84.2%)	49 (94.2%)	
7–18 days, no.	6 (15.8%)	3 (5.8%)	
End time, day	17.0 (8.0, 29.5)	15.5 (7.3, 21.8)	0.57
< 31 days, no.	29	46	
31–61 days, no.	6	4	
> 61 days, no.	2	2	
CCD degree, median (IQR)	2.0 (1.0, 2.0)	1.0 (1.0, 2.0)	0.10
Grade 1, no.	19	34	
Grade 2, no.	18	15	
Grade 3, no.	3	3	
CCD quadrants, median (IQR)	2.0 (1.0, 4.0)	2.0 (1.0, 4.0)	1.00
IOP, mmHg, median (IQR)			
Lowest	6.0 (5.0, 7.8)	6.0 (4.0, 7.0)	0.14
First measure	7.0 (6.0, 9.8)	7.0 (5.5, 9.0)	0.62
Reposition	13.0 (8.0, 15.0)	13.0 (10.0, 16.3)	0.55

*IQR* interquartile range, *IOP* intraocular pressure; all the variables in Table 2 were compared by Mann-Whitney *U* test

By using univariable and multivariable logistic regression analysis, axial length and previous surgical history were found to be associated with the occurrence of CCD (Table 3). For eyes with longer axial length possessed 0.78 times less likely of the happening of CCD. Besides of this, eyes with previous surgical histories were 4.06 times more likely to complicate with CCD. The counted previous surgery included 14 types of intraocular procedures. For the past surgical history, phacoemulsification plus intraocular lens implantation (18 cases), trabeculectomy (15 cases) were the most common in the CCD eyes. While in the Non-CCD eyes, trabeculectomy (7 cases), vitrectomy (5 cases) and retinal photocoagulation (5 cases) were the most frequent procedures (Table 4). The other previous surgical procedures included laser peripheral iridotomy, cyclocryopexy, vitreal injection, goniosynechialysis, anterior chamber paracentesis, corneal suture and cyclophotocoagulation (Table 4). For the success rate, there was no significant difference between CCD and Non-CCD group (Table 5).

**Table 3:** Univariable and multivariable logistic regression showing predictors of CCD

Variable	Univariable		Multivariable	
	Odds Ratio (95% CI)	*p* value	Odds Ratio (95% CI)	*p* value
Age	1.01 (0.99–1.03)	0.32	1.00 (0.97–1.04)	0.83
Gender	0.91 (0.47–1.75)	0.78	1.04 (0.30–3.59)	0.95
Glaucoma diagnosis	1.06 (0.85–1.31)	0.61	0.94 (0.63–1.40)	0.76
Axial length	0.90 (0.79–1.04)	0.15	0.78 (0.62–0.97)	0.02
Surgical type	1.00 (0.53–1.90)	0.98	1.52 (0.40–5.70)	0.54
Baseline medication	0.94 (0.64–1.39)	0.76	1.06 (0.58–1.93)	0.86
Previous surgery	3.33 (1.72–6.45)	< 0.001	4.06 (1.24–13.34)	0.02
Lens status	1.00 (0.83–1.23)	0.93	1.04 (0.67–1.60)	0.88
Baseline IOP	1.00 (0.98–1.03)	0.86	1.00 (0.96–1.05)	0.96

*IOP* intraocular pressure, *CI* confidence interval. All variables were included in the multiple logistic regression)

**Table 4:** Previous surgical procedures comparison

	CCD			Non-CCD		
	Total	AGV alone	AGV-Phaco	Total	AGV alone	AGV-Phaco
Phaco+IOL	18	17	1	4	4	0
Trabeculectomy	15	5	10	7	1	6
Laser peripheral iridoctomy	7	4	3	2	1	1
Cyclocryopexy	7	1	6	1	0	1
Vitreal injection	6	6	0	3	2	1
Goniosynechialysis	5	5	0	0	0	0
Vitrectomy	5	4	1	5	5	0
AC paracentesis	4	3	1	2	1	1
Retinal photocogulation	4	2	2	5	5	0
Undefined anti-glaucoma	4	4	0	3	3	0
Undefined RD reduction	1	1	0	1	1	0
Corneal suture	1	1	0	1	0	1
Cyclophotocoagulation	1	0	1	0	0	0

Phaco+IOL = phacoemulsification plus intraocular lens implantation; *RD* retinal detachment; *AC* anterior chamber

**Table 5:** Success Rate Comparisons

	Success rate	*p* Value	Modified success rate	*p* Value
CCD	64.3%	0.86	73.3%	0.77
Non-CCD	62.5%		71.2%	

Figure 3 illustrated the differences of the IOP at three postoperative moments, namely (1) postoperative day-1, (2) when CCD was firstly detected and (3) the lowest IOP with the presence of CCD. The IOP level of both situations (2) and (3) were significantly lower than that in (1), situation (2) was also significantly lower than situation (3) either in groups of all patients, AGV alone or AGV-Phaco (all *p* <  0.01).

## Discussion

CCD is a common complication after glaucoma filtering surgeries. Previously, our group reported using AS-OCT to investigate CCD after trabeculectomy with its advantages of non-contact scanning and higher resolution than ultrasound biomicroscopy [3]. In this study, we further using AS-OCT to investigate CCD after AGV surgery and it was observed in 57.8% in all patients. This is slightly higher than the rate of 42% after ab interno trabeculotomy and lower than the incidence of 76% after trabeculectomy [1, 3]. This variation may be due to the different surgical procedures and various mechanisms of CCD. However, CCD has no impact on the success rate after AGV surgery at 6 months postoperatively.

**Fig. 2:**
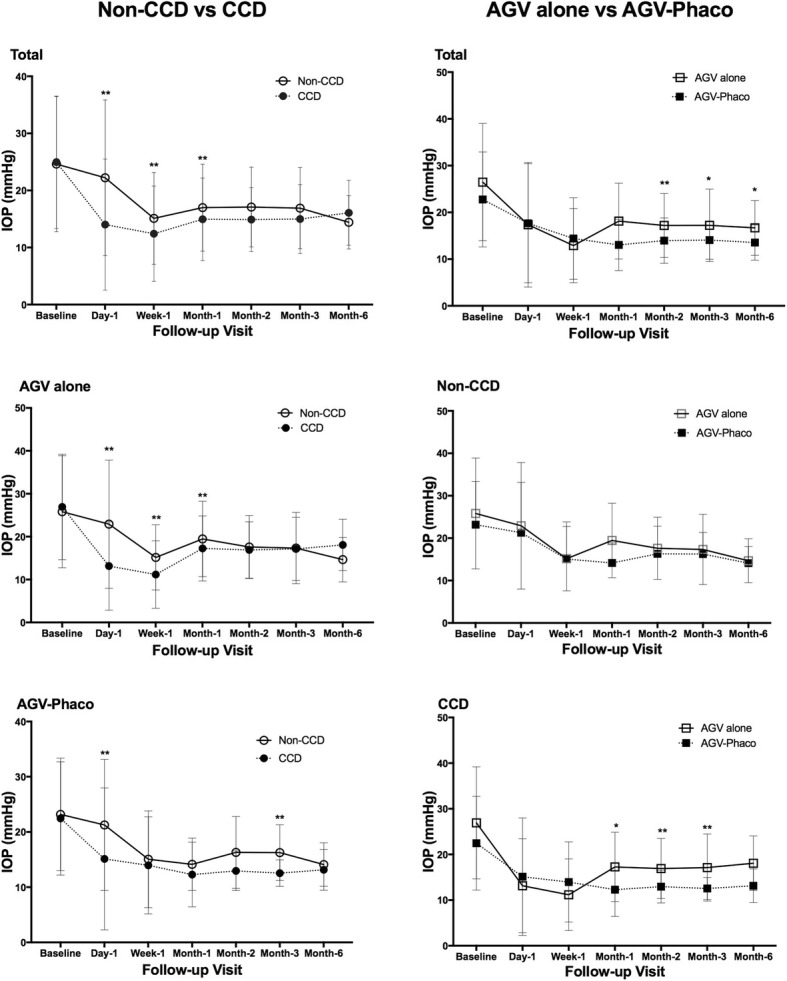
Comparison of IOP in Different Groups. CCD showed differential effect on the postoperative IOP in two surgical types. * *p* < 0.05, **p < 0.01

**Fig. 3:**
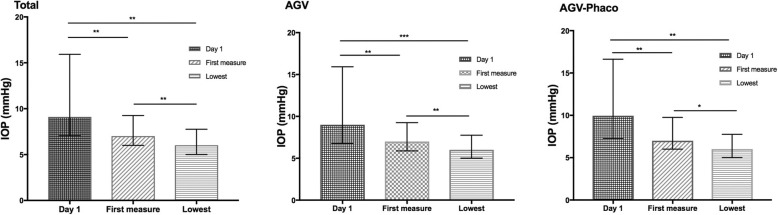
IOP Differences Measured at 3 Different Time Points in 3 Groups. The first measure and lowest IOP were significantly lower than the IOP measured at day-1 postoperatively. * *p* < 0.05, **p < 0.01

In this work, we also wantto identify the possible risk factors of CCD. It is observed that patients with previous surgical history are prone to CCD. The phacoemulsification plus intraocular lens implantation and the trabeculectomy were the two commonest previous surgeries in our study. These two are both incisional procedures which can lower the IOP postoperatively. It was reported that choriocapillary disruption caused by surgical procedures like scleral buckle and panretinal photocoagulation may be the mechanism of CCD [5, 18]. However, this is unlike the situation in phacoemulsification and glaucoma drainage surgery [18, 19]. Transient IOP fluctuation and inflammation induced by phacoemulsification and glaucoma drainage surgery are more likely to be the mechanisms of CCD. Besides of the previous surgical history, we also found the shorter axial length is another predictor of CCD, which is in consistent with results from Akagi T, et al. [1, 18] However, the deep relationship between the axial length and CCD is till unclear. The relationship between IOP and CCD is controversial. CCD was said to be related to the postoperative IOP reduction due to its contribution to the increase of uveoscleral aqueous outflow [1]. From our results, IOP at the postoperative day-1,week-1 and month-1 was lower in the CCD group than in the Non-CCD group (Fig. 2). It indicates that CCD may be associated with the IOP lowering during the first week and first month after surgery. In addition, from the comparison of IOP among postoperative day-1, first measure when CCD occur and lowest IOP during CCD present, IOP after CCD was significant lower than that before the occurrence of CCD (Fig. 3). This means that after CCD happened, IOP continued to decrease. This result agrees with previous report that CCD is related to postoperative IOP lowering [20]. Ocular hypotension was also suggested to be a possible trigger of CCD after glaucoma filtering surgeries due to aqueous humor leakage [3, 20, 21]. In our study, IOP at postoperative day-1 before CCD detected was also lower in the CCD group than the Non-CCD eyes, this suggests that postoperatively, lower IOP may also be a risk factor of CCD. And the IOP range of 5.5 mmHg to 9.8 mmHg from the first measure of CCD patients may give a signal to doctors that CCD will proceed.

The occurrence of CCD on the AGV-Phaco and AGV alone group was not exactly the same. In our observation, 84.2 and 94.2% occurred in less than 1 week in AGV-Phaco and AGV alone surgery respectively. Moreover, CCD in the AGV-Phaco group was observed later than in the AGV alone group. The underlying mechanism is unclear. We suspect that this may be due to that most AGV alone eyes had underwent lens extraction surgery before and was easier to CCD occurrence than AGV-Phaco. It is said cataract extraction induced centripetal movement of ciliary body [22, 23]. Eyes with previous ciliary body centripetal movement may appear CCD earlier than newly ciliary body centripetal movement. Another difference between AGV-Phaco and AGV alone eyes is that the IOP in the total AGV-Phaco group was lower than the AGV alone group from month-2 to month-6 (Fig. 2). In Non-CCD eyes, IOP did not show any differences between two surgical groups. These means the lowering of IOP in the AGV-Phaco group was mainly due to the presence of CCD. It is reported that phacoemulsification and IOL implantation itself can reduce the IOP by 16 to 26% in glaucoma patients [24, 25]. However, in our study, phacoemulsification and IOL implantation itself only showed stable additional IOP lowering effect on the CCD eyes but not on Non-CCD eyes when combined with AGV implantation (Fig. 2). Since most CCD recovered before month-1, it is supposed that there is remained functional interactions between IOL and anatomic recovered CCD which is associated with IOP lowering. While this effect did not influence the surgical success rate between CCD and Non-CCD eyes. Hence, CCD may be not a worrisome complication in controlling IOP after AGV surgery. It may conversely benefit for patient mood control since it showed relative low IOP in early duration after surgery.

There are limitations of this study. This is a retrospective study, which the data of central corneal thickness, an associated factor of CCD [1], as well as visual function evaluations of patients, were not available in this study. And posterior choroidal detachment, which is also related to IOP and visual function, was not regularly looked for in this study. Moreover, a follow-up study with only 6 months is relatively shorter than in common glaucoma studies. Hence a prospective design is urging to evaluate the influence of CCD on not only IOP, but also visual function as well as its long-term effect on the prognosis of patients.

In conclusion, CCD was common after Ahmed glaucoma valve implantation surgery. It is more prone in eyes with previous surgical history and longer axial length may be a protective factor. Nevertheless this complication did not influence the success rate 6 months after surgery.

## Abbreviations

AGV: Ahmed glaucoma valve
AGV-Phaco: combined Ahmed glaucoma valve implantation with phacoemulsification
AS-OCT: anterior segment optical coherence tomography
BCVA: best-corrected visual acuity
CCD: ciliochoroidal detachment
CI: confidence interval
CT: corneal thickness
ICES: iridocorneal endothelial syndrome
IOL: intraocular lens
IOP: intraocular pressure
OR: odds ratio
POAG: primary open angle glaucoma
